# Exosomes and Their Noncoding RNA Cargo Are Emerging as New Modulators for Diabetes Mellitus

**DOI:** 10.3390/cells8080853

**Published:** 2019-08-08

**Authors:** Wenguang Chang, Jianxun Wang

**Affiliations:** 1 Center for Regenerative Medicine, Institute for Translational Medicine, Qingdao University, Qingdao 266021, China; 2 School of Basic Medical Sciences, Qingdao University, Qingdao 266021, China

**Keywords:** exosomes, miRNA, long noncoding RNA, type 1 diabetes, type 2 diabetes

## Abstract

Diabetes belongs to a group of metabolic disorders characterized by long term high blood glucose levels due to either inadequate production of insulin (Type 1 diabetes, T1DM) or poor response of the recipient cell to insulin (Type 2 diabetes, T2DM). Organ dysfunctions are the main causes of morbidity and mortality due to high glucose levels. Understanding the mechanisms of organ crosstalk may help us improve our basic knowledge and find novel strategies to better treat the disease. Exosomes are part of a newly emerged research area and have attracted a great deal of attention for their capacity to regulate communications between cells. In conditions of diabetes, exosomes play important roles in the pathological processes in both T1DM and T2DM, such as connecting the immune cell response to pancreatic tissue injury, as well as adipocyte stimulation to insulin resistance of skeletal muscle or liver. Furthermore, in recent years, nucleic acids containing exosomes—especially microRNAs (miRNAs) and long noncoding RNAs (lncRNAs)—have been shown to mainly regulate communications between organs in pathological processes of diabetes, including influencing metabolic signals and insulin signals in target tissues, affecting cell viability, and modulating inflammatory pancreatic cells. Moreover, exosome miRNAs show promise in their use as biomarkers or in treatments for diabetes and diabetic complications. Thus, this paper summarizes the recent work on exosomes related to diabetes as well as the roles of exosomal miRNAs and lncRNAs in diabetic pathology and diagnosis in order to help us better understand the exact roles of exosomes in diabetes development.

## 1. Extracellular Vesicles and Exosomes

**Figure 1:**
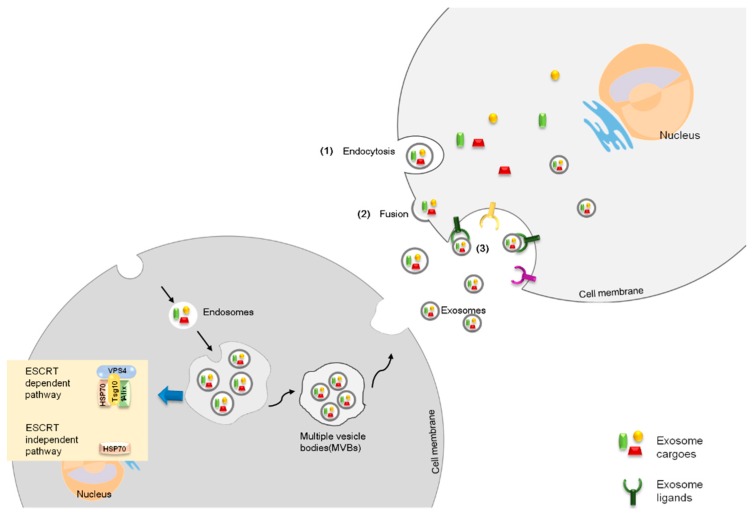
Exosome biogenesis and regulation. Schematic representation of exosome biogenesis and uptake. Exosomes are initially formed as intraluminal vesicles by multivesicular bodies (MVBs). MVB formation has two mechanisms. The endosomal sorting complex required for transport (ESCRT)-dependent pathway requires a complex constituent of ESCRT, Vps4 (vacuolar protein sorting-associated protein), and Hsp70 (heat-shock protein). The typical proteins Tsg101 and Alix for exosome identification belong to the ESCRT complex and represent ESCRT. The other pathway involves ESCRT-independent pathways, which are dependent on Hsp70 binding phospholipids to assemble MVBs. After secretion, the recipient cells mainly uptake those exosomes in three ways: (**1**) endocytosis, by which exosomes enter into the recipient cell cytosol directly; (**2**) fusion, by which the exosome cargo is mainly released to the recipient cytosol; or (**3**) ligands, by which exosomes interact with the recipient cells either by inducing internalization or eliciting intracellular signaling cascades without being internalized.

Communication between organs is crucial for their normal functioning and pathological physiology [1,2,3]. In addition to the traditional known factors that affect organ communication such as cytokines, hormones, and chemokines, a new family involving extracellular vesicles (EVs) has emerged as a group of important modulators. The generic term “extracellular vesicles” was proposed to define all lipid bilayer-enclosed extracellular structures. EVs can be secreted by almost all cell types, including plant cells, bacteria, and fungi [4,5,6,7,8]. These small vesicles can be released in response to various stimulation, for example, changes in cell physical environments (PH, temperature, irradiation) or cell stress and activation induced by chemical agents [9,10,11,12,13]. Accruing literature reveals four major classes of EVs, which are sorted by size, subcellular origin, and content, named microvesicles, apoptotic bodies, virus-like particles, and exosomes [14,15,16,17,18]. The first three types of EVs are formed by outward budding of the plasma membrane. Exosomes, which are unlike other EVs, range from 30 to 100 nm in size and are formed by an intracellular endocytic trafficking pathway involving fusion of multivesicular late endocytic compartments [multivesicular bodies (MVBs)] with the plasma membrane (Figure 1). The generation of exosomes is initially formed as intraluminal vesicles (ILVs) (Figure 1) [19,20]. When an MVB fuses with the plasma membrane, the intraluminal vesicles (also called exosomes) are released to the extracellular space (Figure 1). The molecular mechanisms of MVB formation include two distinct pathways: the endosomal sorting complex required for transport (ESCRT) dependent pathway, and the ESCRT independent pathway. The ESCRT pathway requires formation of a complex by ESCRT, the sorting protein, Vps4, and the constitutive heat-shock protein, Hsp-70 [21,22,23]. The ESCRT is a family of proteins that associate in successive complexes (ESCRT-0, -I, -II, and -III) at the membranes of MVBs to regulate the formation of ILVs as well as their cargo [4]. The typical tumor susceptibility gene 101 protein (Tsg101) and Alix (encoded by PDCD6IP) for exosome identification belong to the ESCRT complex. The other pathway involves ESCRT-independent pathways. This pathway regulates the assembly of exosomes and requires Hsp70-phospholipid interactions and the activity of acid sphingomyelinase (nSMase), an enzyme that hydrolyzes sphingomyelin without the presence of ESCRTs [24,25] (Figure 1).

## 2. Exosome Uptake

Exosomes carry diverse cargo molecules, such as signal lipids, functional proteins, and nuclei [26,27,28,29,30], which can mediate intercellular communications by initiating a range of biological responses by acceptor cells. Upon secretion, those exosomes are released into the extracellular space and incorporated with the body fluids. Concurrently, the recipient cells uptake those exosomes through various processes. An exosome can either enter the recipient cell cytosol via phagocytosis/endocytosis or fuse with the recipient plasma membrane and directly release the cargo (Figure 1). During exosome biogenesis, diverse surface proteins are displayed on exosomes and recipient cells, including integrins, lectins/proteoglycans, and T cell immunoglobulin and mucin domain containing protein 4 (Tim4) [31,32]. Integrin plays a crucial role in the recognition of target recipient cells [33,34]. Therefore, exosomes can also interact with the recipient cells via the ligands on the cell surface in a protein–protein interaction, induce internalization, or promote intracellular signaling cascades without being internalized (Figure 1). Exosomes are responsible for a variety of normal physiological processes and abnormal pathological processes, such as the progression of neuronal degeneration [35,36], cancer [37,38], and diabetes.

## 3. Exosomes and Diabetes

Diabetes is part of a group of metabolic disorders characterized by long-term high blood glucose levels due to either inadequate production of insulin (type 1) or poor response of the recipient cell to insulin (type 2) [39]. According to the International Diabetes Federation (IDF), in 2017, 451 million people (aged 18–99 years) had diabetes worldwide. This number is projected to increase to 693 million by 2045 globally [40]. Thus far, the exact pathology of diabetes is still not fully understood. Studies have shown that the mechanisms underlying diabetes are complex and may involve changes at both the organ and the gene expression levels [41,42,43].

### 3.1. Exosomes and Type 1 Diabetes

**Figure 2:**
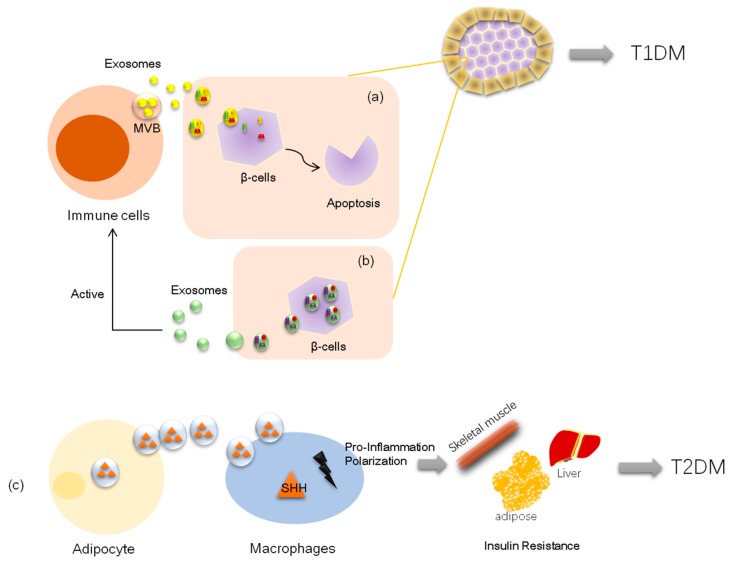
Exosomes regulate diabetic pathological process. Schematic representation of exosomes participating in the pathologies of type 1 diabetes (T1D) and type 2 diabetes (T2D). (**a**) Immune cell-derived exosomes can activate immune cells and induce β-cell apoptosis and cell death. (**b**) In certain models, pancreatic islets also release intracellular β-cell autoantigens in exosomes in turn to favor the immune response. (**c**) Adipocyte-derived exosomes carrying sonic hedgehog (SHH), a protein known to modulate immunity, induce proinflammatory or M1 polarization of bone marrow-derived macrophages and induce insulin resistance in the main insulin-sensitive organs (skeletal muscle, liver, adipose).

The pathology of type 1 diabetes (T1DM) is known to be related to autoimmune disorders. A complex interaction between pancreatic β-cells and an innate or an adaptive immune system leads to the irreversible destruction of insulin-producing cells in pancreatic islets [44,45,46,47]. With more in-depth research, a relationship between exosomes and type 1 diabetes has emerged in recent years [48]. On one hand, exosomes carry active immune molecules (normally proteins or nuclei) that can activate immune cells, such as T cells and B cells, and induce β-cell apoptosis, thereby contributing to T1DM development [49,50] (Figure 2). On the other hand, researchers found that rat and human pancreatic islets also release intracellular β-cell autoantigens in human T1DM, GAD65, IA-2, and proinsulin in exosomes, which are taken up by and activate dendritic cells [13]. Moreover, insulinoma-derived exosomes stimulate the innate immune response in the Myd88-dependent pathway, which is an inflammatory signaling downstream of members of the Toll-like receptor (TLR) and the interleukin-1 (IL-1) receptor families [51], and exosomes derived from islet mesenchymal stem cell (MSCs) directly activate the T cell response and stimulate the release of interferon gamma (IFN-γ) to induce inflammation, which plays a role in the initiation of autoimmune responses in T1D [52,53] (Figure 2). In addition, some exosomes are promising as effective and practical candidates for T1D therapy. For instance, adipocyte-derived stem cell exosomes exert ameliorative effects on T1DM by multiple pathways, including modulating the immune cell response [54], attenuating podocyte damage [55], and promoting proangiogenic properties [56]. Nonetheless, exosomes can be developed as an effective tool to improve islet transplant by modulating the immune response or as a biomarker of recurrent autoimmunity for islet transplant diagnosis [57,58].

### 3.2. Exosomes and Type 2 Diabetes

Type 2 is the main phenotype of diabetes, accounting for more than 90% of the diabetic population [59]. It is characterized by high plasma glucose due to insulin resistance. Excessive accumulation of fat due to obesity in adipose tissue has been considered a major driver in the pathophysiology of insulin resistance [60,61,62]. Recent studies have shown that adipocytes can produce and release exosomes containing bioactive cargo and acting as a mechanism of cell-to-cell communication, which contributes to the incidence of insulin resistance. For example, adipocyte-derived exosomes carrying sonic hedgehog (SHH), a protein known to modulate immunity, induce proinflammatory or M1 polarization of bone marrow-derived macrophages and RAW 264.7 macrophages via the Ptch/PI3K signaling pathway and contribute to insulin resistance [63] (Figure 2). Additionally, exosomes derived from insulin-resistant adipocytes promote plaque burden and plaque vulnerability partly by inducing vasa vasorum angiogenesis in human umbilical vein endothelial cells [64] (Figure 2). In addition to adipocytes, exosomes derived from other cells or organs also contribute to insulin resistance, including bone marrow mesenchymal cells, human placental cells, and cardiomyocytes [65,66,67,68]. Moreover, exosomes are promising as a therapeutic agent for type 2 diabetes. Recent studies have shown that exosomes derived from umbilical cord MSCs relieve β-cell destruction and alleviate type 2 diabetic nephropathy [69,70]. Another investigation also related exosomes to the mechanism underlying the beneficial effects of exercise or bariatric surgery on type 2 diabetes [71,72,73].

## 4. Exosome Noncoding RNA in Diabetes

Exosomes secreted from different cells participate in the progression of diabetes in distinguishing ways; however, the role of exosomes not only depends on the origin but also is mainly decided by the cargo content. In recent years, exosome nucleic acids, especially noncoding RNA-containing exosomes, have been shown to be involved in a wide range of processes that underlie diabetic progression; those noncoding RNAs have been widely studied and were found to exert their functional roles in remote tissues, which indicated important roles in multiple biological functions through diverse mechanisms, including recruitment of epigenetic modifier proteins, control of mRNA decay and translation, and DNA sequestration of transcription factors [74,75].

### 4.1. MiRNA in Exosomes and Their Roles in Diabetes

MicroRNAs (miRNAs) are small, noncoding RNA molecules containing approximately 22 nucleotides. miRNAs are found in plants, animals, and some viruses that function in RNA silencing and posttranscriptional regulation of gene expression [76]. Many microRNAs, such as let-7, miR-223, miR-29, miR-103, and miR-107, are known to regulate metabolic disorders (including diabetes) through various molecular pathways, such as modulation of lipid or glucose metabolism, liver gluconeogenesis, insulin secretion, and autophagy [77,78,79,80,81]. MiRNA-carrying exosomes have advantages that work at distant tissues more efficiently. Thus, some exosomal miRNAs are pathological factors in diabetes by targeting key proteins and acting as crucial modulators of insulin sensitivity. In cells, insulin combined with its substrate, insulin receptor substrate-1 (IRS-1), stimulates a cascade signal, including phosphorylation of protein kinase B (PKB/AKT), at serine 473, leading to phosphorylated AKT promoting the translocation of glucose transporter-4 (Glut4) from the cytosol to the membrane for glucose uptake [82]. Katayama and colleagues examined the miRNA expression profile of serum-derived exosome-enriched extracellular vesicles in healthy controls and type 2 diabetic patients. They found that exosome-derived extracellular miR-20b-5p is highly abundant in type 2 diabetic patients, and further in vitro studies showed that exosomal miR-20b-5p targeted AKT-interacting protein (AKTIP), which interacts directly with AKT and modulates AKT activity by enhancing the phosphorylation of AKT regulatory sites and reducing glycogen accumulation in primary human skeletal muscle, resulting in insulin resistance [83]. Consistent with the above findings, another study demonstrated that pancreatic cancer-derived exosomes enter C2C12 myotubes and inhibit insulin and PI3K/Akt signaling, thereby preserving insulin-induced FoxO1 nuclear exclusion and impairing Glut4 trafficking. In this study, microarray methods revealed that certain exosomal microRNAs (miR-450b-3p and miR-151-3p) likely play key roles in this process (Figure 1) [84]. Further, exosome miR-155 derived from adipose tissue macrophages in obesity-induced diabetic mice causes glucose intolerance and insulin resistance by targeting PPARγ, a transcription factor and key regulator of lipid metabolism, when administered to lean mice [85]. Similarly, another elegant experiment on obese mice showed that exosomes from obese mice could induce glucose intolerance in lean mice; in this study, the authors transfected exosomes with the selected miRNA (miR-192, miR-122, miR-27a-3p, and miR-27b-3p; all were demonstrated to be increased in obese mice) and then injected the reconstituted exosomes into lean mice. Surprisingly, the lean mice developed glucose intolerance and insulin resistance as well. The data further showed that exosomal miRNAs induced diabetes in white adipose tissues in lean mice by targeting PPARα [86], which is also a transcription factor and a major regulator of lipid metabolism.

Inadequate pancreatic β-cell numbers is a pathological manifestation of type 1 diabetes. Researchers have found that lymphocyte-derived exosomes miR-142-3p and miR-142-5p from prediabetic mice transferred to pancreatic β-cells trigger the expression of genes involved in chemokine signaling, including Ccl2, Ccl7, and Cxcl10. The induction of these genes may promote the recruitment of immune cells and exacerbate β-cell death during an autoimmune attack, which also contributes to diabetes development [49]. Nonetheless, a recent study demonstrated that bone marrow mesenchymal stem cells derived exosome miR-29b-3p from aged mice mediated insulin resistance in young mice [65], which indicated that exosomal miRNAs could exert their role independently. Thus, exosome cargo with miRNAs is also considered a novel therapeutic agent. Exosome miR-222 was reduced in rabbits with type 1 diabetes; however, MSC-derived exosomes containing miR-222 injected subconjunctivally and intraocularly to these diabetic rabbits could attenuate retinal degeneration [87]. Similarly, intravenous administration of exosome miR-106b and miR-222 derived from bone narrow promoted post-injury β-cell proliferation through Cip/Kip family downregulation in insulin-deficient mice [88]. In addition, MSC-derived exosome miR-146a has anti-inflammatory effects on damaged astrocytes and prevents diabetes-induced cognitive impairment [89].

Consequently, many exosomal miRNAs have been found to be increased in the plasma or the urine of diabetic patients, making them promising circulating biomarkers associated with type 2 diabetes, such as exosomal miR-21-5p, miR-375-3p, miR-133b, miR-342, and miR-30, which are all upregulated in the serum of diabetic subjects [90,91,92], and miR-451-5p, let-7c-5p, miR-362-3p, miR-877-3p, miR-150-5p, and miR-15a-5p, which are upregulated in the urine of diabetic subjects [93,94,95]. It should be noted that, for certain miRNAs, total circulating miRNA levels are distinct from circulating extracellular vesicle miRNA content. For example, a clinical study including 19 children with type 1 diabetes along with 16 healthy controls showed that miR-21-5p derived from serum extracellular vesicles was increased threefold compared with that in nondiabetic individuals, while total serum miR-21-5p was reduced in diabetic participants [92], suggesting that exosomal RNA may be a distinct biomarker from total serum RNA levels. These data are summarized in Table 1.

### 4.2. Other Noncoding RNAs in Exosomes and Their Roles in Diabetes

In addition to miRNA, other noncoding RNAs, such as circular RNA (circRNAs), P-element induced Wimpy testis (PIWI)-interacting RNAs (piRNAs), and long noncoding RNA (lncRNAs), are also known to participate in the pathological process of diabetes. CircRNA is a type of noncoding RNA that forms a unique, circular, covalently closed continuous loop. This kind of RNA is resistant to exonuclease-mediated degradation because it does not have 5’ or 3’ ends and is presumably more stable than most linear RNAs. Recent evidence has shown that circRNAs usually regulate the transcription of miRNA-target genes and function as miRNA sponges. Several circRNAs have been implicated in the pathological progression of diabetes, including reducing β-cell proliferation, reducing survival, and affecting insulin secretion [96,97,98]. PiRNAs belong to another class of small RNAs that are 24–31 nucleotides in length. They were first found in germline cells and play a key role in spermatogenesis. PiRNAs interact with PIWI proteins and guide them to silence transposable elements, therefore silencing gene expression, regulating mRNA translation, or sustaining stem cell stability. Recently, researchers found that piRNAs are also expressed in pancreatic islets and participate in the control of β-cell activities [99]. However, although circRNAs and piRNAs are known to exist in exosomes, there is still a lack of evidence showing exosomal circular RNA or piRNA changes in diabetic state.

**Figure 3:**
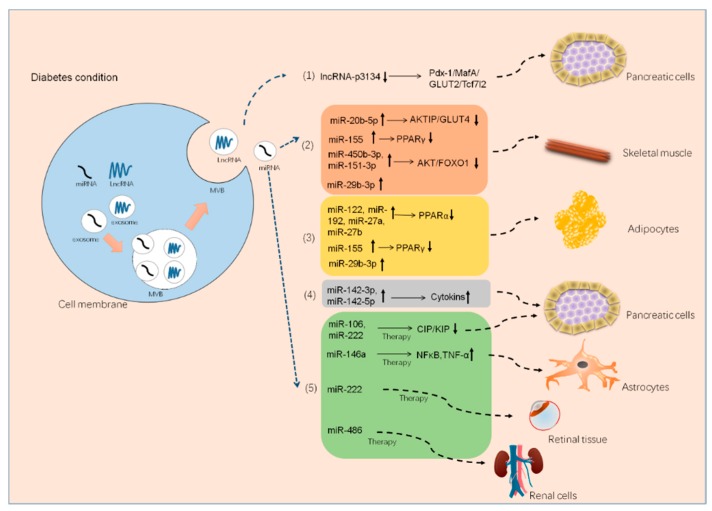
The molecular mechanisms by which exosomes containing miRNA and long noncoding RNA (lncRNA) regulate communication between organs. In the diabetic state, miRNA- and lncRNA-carrying exosomes exert their biological function by targeting different tissues. (**1**) Exosome lncRNA-p3134 was downregulated in a diabetic model, and lncRNA-p3134 could positively regulate glucose-stimulated insulin secretion by promoting key regulators (Pdx-1, MafA, GLUT2, and Tcf7l2) in β-cells. In this case, downregulation of lncRNA-p3134 contributed to the pathology of diabetes. (**2**) Exosomal miRNA (miR-20b-5p, miR-155, miR-450b-3p, miR151-3p, and miR-29b-3p) was upregulated in the diabetic model and targeted skeletal muscles via various insulin signaling regulatory proteins (PPAR, AKT, GLUT4, and FOXO) and then contributed to the pathology of diabetes. (**3**) Exosomal miRNA (miR-122, miR-192, miR-27a/b, miR-155, miR-29b-3p) was upregulated in diabetic models and targeted adipocytes via PPAR proteins and then contributed to the pathology of diabetes. (**4**) Exosomal miRNA (miR-142-3p and miR-142-5p) was upregulated in diabetic models and targeted pancreatic cells via upregulated cytokines, which then contributed to the pathology of diabetes. (**5**) Other exosomal miRNAs (miR-106, miR-146a, miR-222, and miR-486) are promising therapeutic agents to protect pancreatic cells or treat diabetic complications by targeting certain organs, such as astrocytes, retinal tissue, and renal cells.

LncRNA is a kind of nonprotein-coding RNA comprising transcripts longer than 200 nucleotides. LncRNA regulates the expression of genes at epigenetic, transcriptional, and posttranscriptional levels and is similar to miRNA. In recent decades, lncRNAs such as ANRIL, H19, MALAT1, Sox2OT, and MEG3 have been implicated in playing important roles in the pathology of diabetes and all are known to associate with the pathology of type 2 diabetes [100,101,102,103,104]. Although only a few studies related exosomal lncRNAs to diabetes, a recent study did show that, although lncRNA-3134 was nearly unchanged in exosome-free samples, lncRNA-p3134 was increased four-fold in serum exosomes, and the circulating level of lncRNA-p3134 was correlated with fasting blood glucose and HOMA-β levels. In a further study, the authors found that the secretion of lncRNA-p3134 positively regulated glucose-stimulated insulin secretion by promoting key regulators (Pdx-1, MafA, GLUT2, and Tcf7l2) in β-cells, and that the upregulation of lncRNA-p3134 in db/db mice restored insulin synthesis and secretion, suggesting lncRNA-p3134 as a compensatory factor to preserve the β-cell function response to high glucose stimulation [105] (Figure 3).

As exosomes have a double membrane structure, they can protect encapsulated small RNAs from ribonucleases (RNases) in body fluid and have low antigenicity and toxicity [106]. In this case, extracellular vesicles, which include exosomes, are considered ideal carriers for nucleic acid drugs. An extracellular vesicle-mimetic nanovesicle containing lncRNA-H19 is considered an effective treatment that could remarkably accelerate the healing processes of chronic wounds induced by diabetes [107], indicating that noncoding RNA-containing exosomes can be a promising candidate for drug delivery.

## 5. Limitations and Conclusions

Current research examining the role of exosomes carrying noncoding RNAs in diabetes is still in its infancy, especially that involving lncRNAs, circular RNAs, or piwi-interacting RNAs. Additionally, in the current research, there are still no clear characterizations, and markers are available for exosomes from different cell types, making it difficult to determine the origin of exosomes in circulation [108,109]. In addition, although exosomes as drug vehicles have been investigated widely, there are still some obstacles in developing exosome-based drug delivery systems [110], such as encapsulation efficiency of cargo, selection of appropriate exosome origination cells, drug loading methodology [111], and exosome surface modification [112,113,114]. However, exosomal miRNA and lncRNA have been shown to modulate diabetic progress in various ways, including influencing the metabolic and the insulin signals in target tissues, affecting cell viability, and modulating inflammation in pancreatic cells. With increasing evidence, exosomes definitely appear to have promising and important roles not only in pathogenesis but also in diabetic diagnosis and therapeutics.

## Figures and Tables

**Table 1:** Current studies of exosomal microRNAs (miRNA) in diabetes.

Exosome MiRNA Name	Main Indication for Diabetes	Expression in Diabetic Statue	Exosome Extracted From	Effected Cells	Experimental Model	Reference
MiR-20b-5p	Pathological factor	upregulate	Serum	Primary human skeletal muscle cells	T2D patients	Katayama, M et al. 2018. [83]
MiR-122,miR-192,miR-27a-3p, miR-27b-3p	Pathological factor	upregulate	Serum	White adipose tissue	Diet-induced obesity mice	Castano, C et al. 2018 [86]
MiR-142-3p, miR-142-5p, miR-155	Pathological factor	upregulate	Lymphocyte	Pancreatic β cells	Non-obese diabetic mice	Guay, C et al. 2018 [49]
MiR-155	Pathological factor	upregulate	Adipose tissue macrophage	Liver, adipose tissue, and muscle	Obese mice	Ying, W et al. 2017 [85]
MiR-29b-3p	Pathological factor	upregulate	Bone marrow mesenchymal stem cells	Adipocytes, C2C12 myocytes, and primary cultured hepatocytes	Aging-related insulin resistance mice	Sun, T et al. 2019 [65]
MiR-486	Therapy agent	upregulate	Adipose derived stem cells	Podocyte cells	Spontaneous diabetic mice	Jin, J et al. 2019 [55]
MiR-222	Therapy agent	downregulate	Adipose tissue mesenchymal stem cells	Retinal tissue	T1D rabbit	Safwat, A et al. 2018 [87]
MiR-146a	Therapy agent	downregulate	Bone narrow stem /stromal cell	Astrocytes	T1D mice	Kubota, K et al. 2018 [89]
MiR-106b, miR-222	Therapy agent	downregulate	Bone narrow stem cell	Pancreatic β cells	T1D mice	Tsukita, S et al. 2017 [88]
MiR-21-5p	Biomarker	upregulate	Serum	NR	T1D patients	Lakhter, A et al. 2018 [92]
MiR-375-3p	Biomarker	upregulate	Serum	NR	New-onset T1D and T2D patients	Fu, Q et al. 2018. [91]
MiR-133b, miR-342, miR-30	Biomarker	upregulate	Serum	NR	T2D patients	Eissa, S et al. 2017 [90]
MiR-451-5p	Biomarker	upregulate	Urinary	NR	T1D rats	Mohan, A et al. 2016 [94]
Let-7c-5p	Biomarker	upregulate	Urinary	NR	T2D patients with kidney disease	Li, W et al. 2018 [93]
MiR-362-3p, miR-877-3p, miR-150-5p, miR-15a-5p	Biomarker	upregulate	Urinary	NR	T2D patients with kidney disease	Xie, Y et al. 2017 [95]

T1D, type 1 diabetes; T2D, type 2 diabetes; NR, not reported.
